# Thyroid hormone activated upper gastrointestinal motility without mediating gastrointestinal hormones in conscious dogs

**DOI:** 10.1038/s41598-021-89378-y

**Published:** 2021-05-11

**Authors:** Nobuhiro Nakazawa, Makoto Sohda, Kyoichi Ogata, Seded Baatar, Yasunari Ubukata, Kengo Kuriyama, Keigo Hara, Masaki Suzuki, Toru Yanoma, Akiharu Kimura, Norimichi Kogure, Akihiko Sano, Makoto Sakai, Takehiko Yokobori, Atsushi Oue, Erito Mochiki, Hiroyuki Kuwano, Ken Shirabe, Noriyuki Koibuchi, Hiroshi Saeki

**Affiliations:** 1 Department of General Surgical Science, Gunma University Graduate School of Medicine, Maebashi, Gunma Japan; 2 Department of Surgery, Kiryu Kosei General Hospital, Kiryu, Gunma Japan; 3 Department of Surgery, Gunma Prefectural Cancer Center, Ota, Gunma Japan; 4 Department of Surgery, Daiichi Hospital, Takasaki, Gunma Japan; 5 Division of Integrated Oncology Research, Gunma University Initiative for Advanced Research (GIAR), Maebashi, Gunma Japan; 6 Department of Bioresource Center, Gunma University Graduate School of Medicine, Maebashi, Gunma Japan; 7 Department of Digestive Tract and General Surgery, Saitama Medical Center, Saitama Medical University, Kawagoe, Saitama Japan; 8 Department of Integrative Physiology, Graduate School of Medicine, Gunma University, Maebashi, Gunma Japan

**Keywords:** Physiology, Gastroenterology

## Abstract

This study was conducted to clarify the relationship between thyroid function and gastrointestinal motility. We established an experimental configuration in which the feedback of thyroid function was completely removed using conscious dogs. With hypothyroidism, time of phase I of interdigestive migrating contractions (IMC) was longer, time of phase II and phase III was significantly shortened, and both the continuous time of strong tetanic contraction at antrum and 10-h frequency of phase III counted from the first IMC after meal significantly decreased. Whereas, hyperthyroidism caused the opposite events to those with hypothyroidism. Furthermore, We found giant migrating contractions (GMC) occurred from the upper gastrointestinal tract when we administrated high dose of thyroid hormone. One GMC occurred from anal sides propagated to cardiac, and this propagation was similar to the emesis-like interdigestive motor activity, the other GMC occurred from oral sides propagated to anal sides and this was similar to the diarrhea-like interdigestive motor activity. We examined the relationship between thyroid function and gastrointestinal hormones including of ghrelin, GLP-1, and cholecystokinin (CCK). However, we could not find significant differences under different thyroid hormone status. This is the first report that thyroid hormone activated upper gastrointestinal motility without mediating gastrointestinal hormones.

## Introduction

Thyroid disease is common and it affects the gastrointestinal (GI) system. In hypothyroidism, the most common GI symptoms are constipation and anorexia. Meanwhile, diarrhea and increased appetite are often observed in hyperthyroidism. Previous studies showed that intestinal transit was shortened in a hyperthyroid state^1–4^. However, there were no changes in gastric emptying, pancreatic secretion, and biliary secretion^5^. The effect of thyroid function on gastric emptying time and GI transit time is controversial. Some reports revealed differences in these functions based on thyroid function^6–12^. Nevertheless, other reports showed that there were no changes^5,13^. To validate this controversy, GI motility must be assessed using electrogastrography. We have previously shown that force transducers detect GI motility^14–35^. Therefore, in this study, we used chronically implanted force transducers to evaluate GI motility.

Gastrin influences GI motility based on thyroid function. Specifically, in hyperthyroidism, serum gastrin concentrations increase. Meanwhile, it decreases in hypothyroidism^36,37^. However, several studies have shown differences in serum ghrelin concentrations based on thyroid function^38–41^. A previous study showed the serum ghrelin concentrations changed based on the phase of interdigestive migrating contractions (IMCs)^20^. IMCs are observed every 90–120 min in both dogs and humans^42,43^. Furthermore, the physiological function of gastric IMC is associated with the mechanical and chemical cleansing of an empty stomach in preparation for the next meal^44^. Regarding the cause of the variation, we considered that none of the previous studies evaluated the phase of IMC. In addition, there was no study on serum glucagon-like peptide-1 (GLP-1) and cholecystokinin (CCK) concentrations according to thyroid function. Hence, the current study assessed these parameters.

This study aimed to validate the relationship between GI motility and hormones under completely eliminating feedback according to thyroid function in conscious dogs.

## Results

### Changes in thyroid hormone levels

The results were consistent in all dogs. That is, hypothyroidism induced low T4 and high thyroid-stimulating hormone (TSH) levels, and hyperthyroidism caused high T4 and low TSH levels, as shown in Table 1. Hence, thyroidectomy and thyroid hormone administration had a stable effect.

**Table 1:** The serum concentrations of T4 and TSH were shown in all conscious dogs.

	T4 (µg/dl)	TSH (ng/ml)
**Dog 1**		
Control	1.7	0.04
Hypothyroidism	< 0.3	0.51
Hyperthyroidism	> 15.0	< 0.02
**Dog 2**		
Control	1.8	0.05
Hypothyroidism	0.5	1.19
Hyperthyroidism	> 15.0	< 0.02
**Dog 3**		
Control	0.97	0.07
Hypothyroidism	< 0.3	2.64
Hyperthyroidism	> 15.0	0.03

### Interdigestive migrating contractions

**Figure 1:**
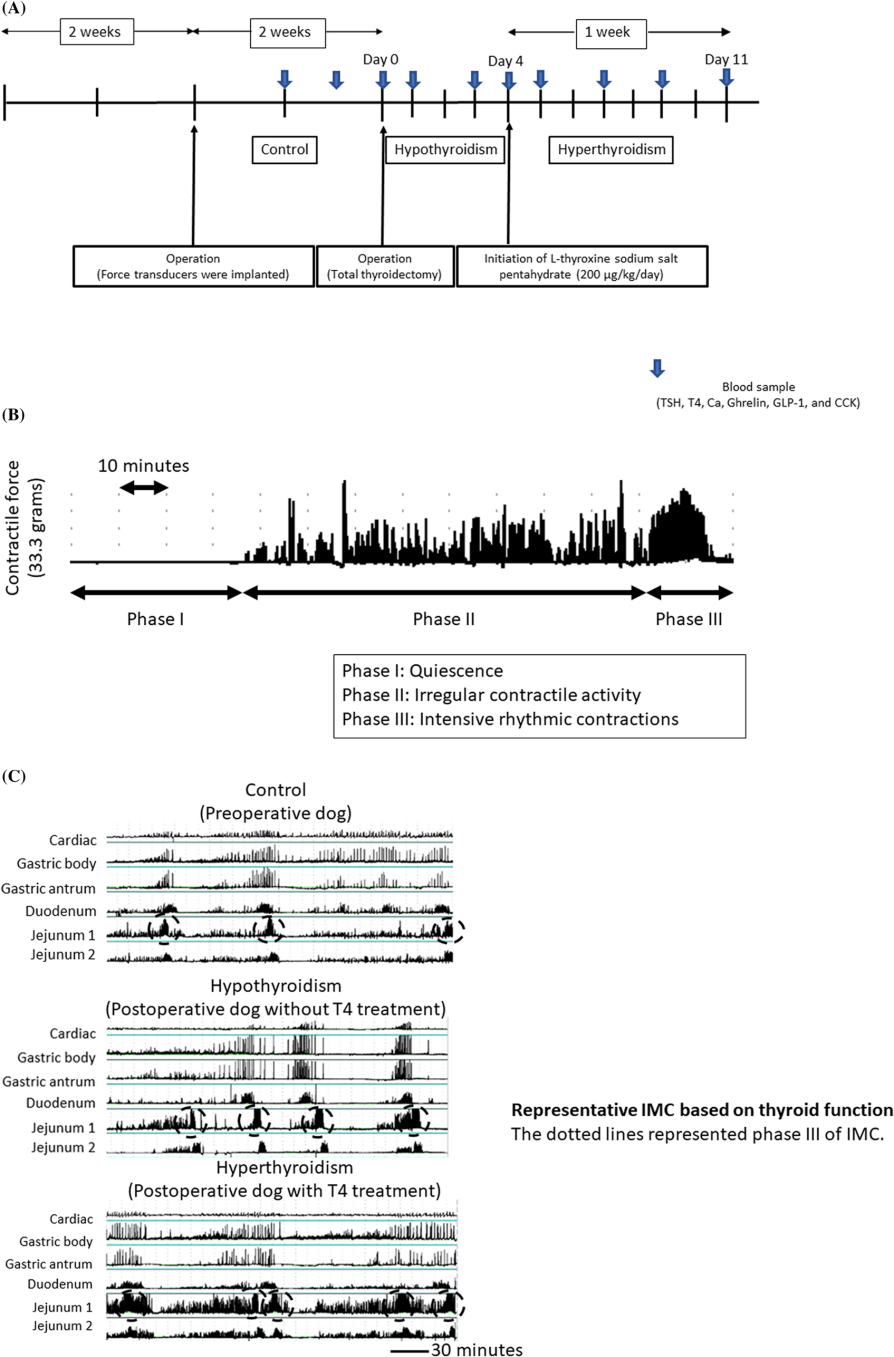
(**A**) Schematic diagram showing the experimental schedule. (**B**) The representative interdigestive migrating contraction (IMC) composed of three phases. Data were obtained from a dog prior to surgery. (**C**) The representative IMC based on thyroid function was presented. The dotted lines represented IMC phase III.

The representative IMC of a preoperative dog is shown in Fig. 1B. The IMC comprised three phases, which are as follows: phase I, quiescence and is divided into the early and late subphases at the midpoint; phase II, irregular contractile activity; and phase III, intensive rhythmic contractions.

### Changes in gastrointestinal motility contractions in response to thyroid function and transmission speed between jejunums 1 and 2

**Figure 2:**
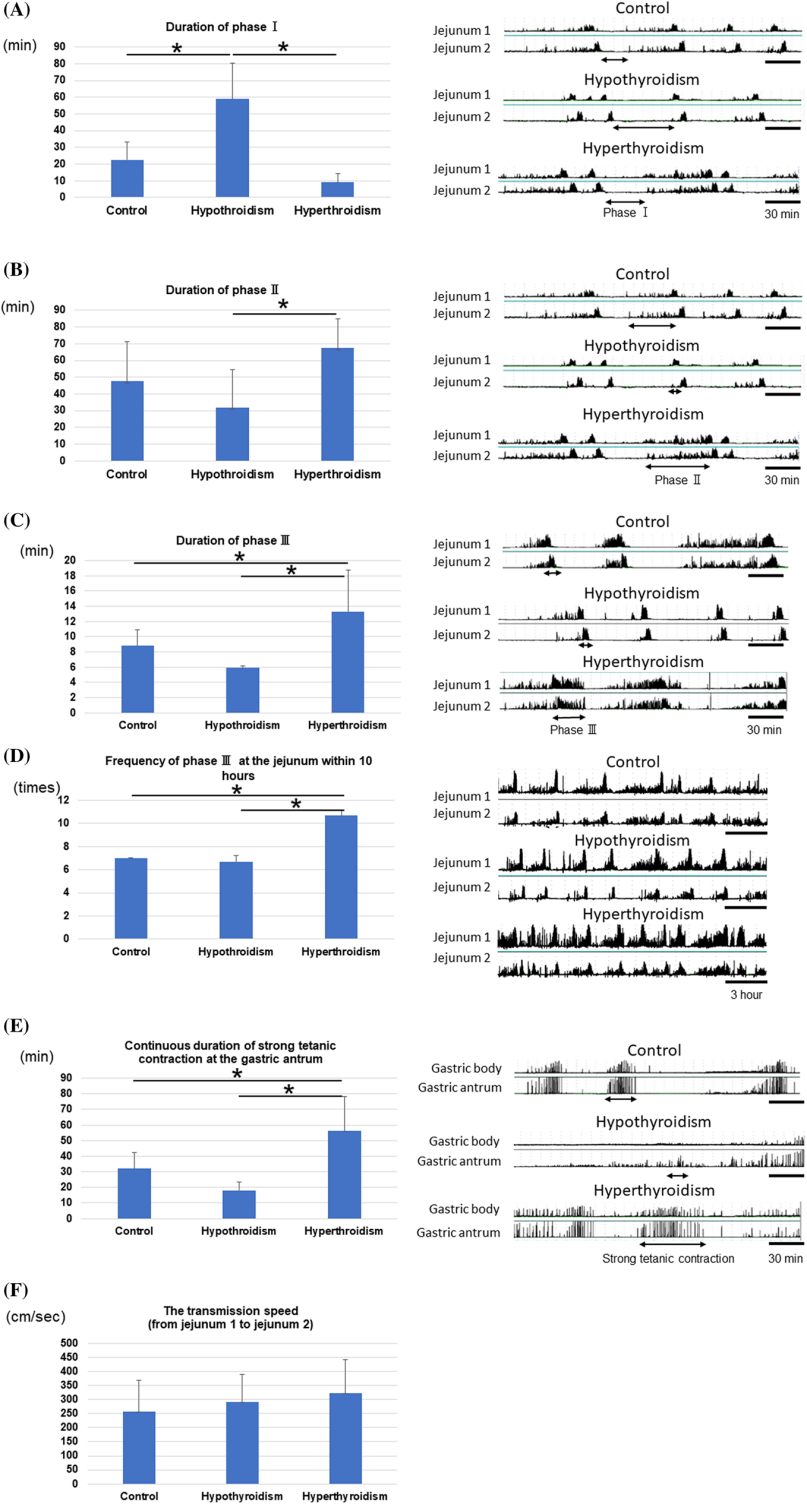
Results of various evaluations about IMC according to thyroid function. (**A**) In a hypothyroid state, the duration of phase I was significantly longer. (**B**,**C**) In a hyperthyroid state, the duration of phases II and III was significantly longer. (**D**) The frequency of phase III at jejunum 1 within 10 h increased under a hyperthyroid state. (**E**) The continuous duration of strong tetanic contractions at the antrum under a hyperthyroid state was significantly longer. (**F**) The transmission speed from jejunum 1 to jejunum 2 did not change regardless of thyroid function.

GI motility contractions according to thyroid function in all dogs are depicted in Fig. 1C. Consistent results were obtained, and the detailed analysis results are presented in Fig. 2. The duration of phase I was significantly longer in hypothyroid states than in control and hyperthyroid states (Fig. 2A). Meanwhile, the duration of phase II was significantly longer in a hyperthyroid state than in a hypothyroid state (Fig. 2B). Furthermore, the duration of phase III was significantly prolonged in a hyperthyroid state compared with control and hypothyroid states (Fig. 2C). The 10-h frequency of phase III counted from the first IMC after a meal was significantly more common in a hyperthyroid state than in both control and hypothyroid states (Fig. 2D). Furthermore, the continuous duration of strong tetanic contractions at the gastric antrum significantly increased in a hyperthyroid state compared with control and hypothyroid states (Fig. 2E). Finally, the evaluation results for transmission speed in control, hypothyroid, and hyperthyroid states are presented in Fig. 2F. However, the results were not significant in this study.

### Changes in gastrointestinal motility contractions before and after thyroid hormone was administered

**Figure 3:**
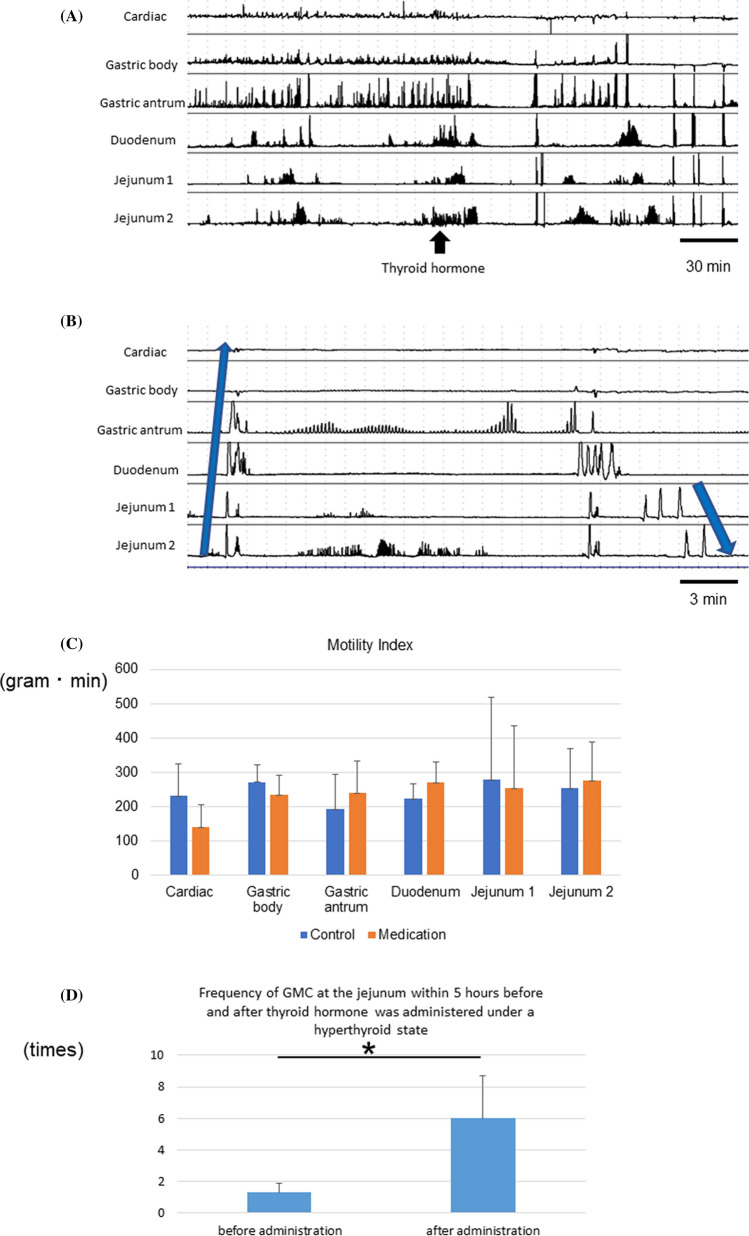
Interdigestive motor activity observed after the intravenous administration of thyroid hormone. (**A**) Representative changes in GI motility contractions before and after thyroid hormone was administered under a hyperthyroid state. Giant migrating contractions (GMCs) were observed after the administration of total thyroxine. (**B**) GMC occurred from both the oral and anal sides. One GMC occurred from the anal sides and propagated to the cardiac side, and this propagation was similar to the emesis-like interdigestive motor activity. The other GMC occurred from the oral sides and propagated to the anal sides, and this propagation was similar to the diarrhea-like interdigestive motor activity. (**C**) Changes in motility index (MI) 2 h before and after thyroid hormone was administered. No significant differences were found in any positions. (**D**) Frequency of GMC at jejunum 1 within 5 h before and after the administration of thyroid hormone. It significantly increased after the administration of thyroid hormone.

We evaluated the changes in days 1, 3, and 5. We administered thyroid hormone at a dose of 200 μg/kg/day. The representative changes in GI motility contractions before and after thyroid hormone was administered are shown in Fig. 3A. Thyroid hormone was administered during fasting. Giant migrating contraction (GMC) was defined as the occurrence of a single large-amplitude contraction. GMC has a larger amplitude and longer duration than a normal contraction^45^. These contractions were observed in all dogs after thyroid hormone was administered. One GMC occurred from the anal sides and propagated to the cardiac sides, and this propagation was similar to the emesis-like interdigestive motor activity in Fig. 3B. The other GMC occurred from the oral sides and propagated to the anal sides, and this was similar to the diarrhea-like interdigestive motor activity.

The MI changes 2 h before and after thyroid hormone was administered are depicted in Fig. 3C. No significant differences were found regardless of the positions of the force transducers. However, the MI decreased in the fornix and gastric body and increased in the gastric antrum and beyond. Moreover, we assessed the frequency of GMC at the jejunum 5 h before and after thyroid hormone was administered under a hyperthyroid state, as shown in Fig. 3D. Results showed that it significantly increased after thyroid hormone was administered.

### Changes in the levels of ghrelin, GLP-1, and CCK

**Figure 4:**
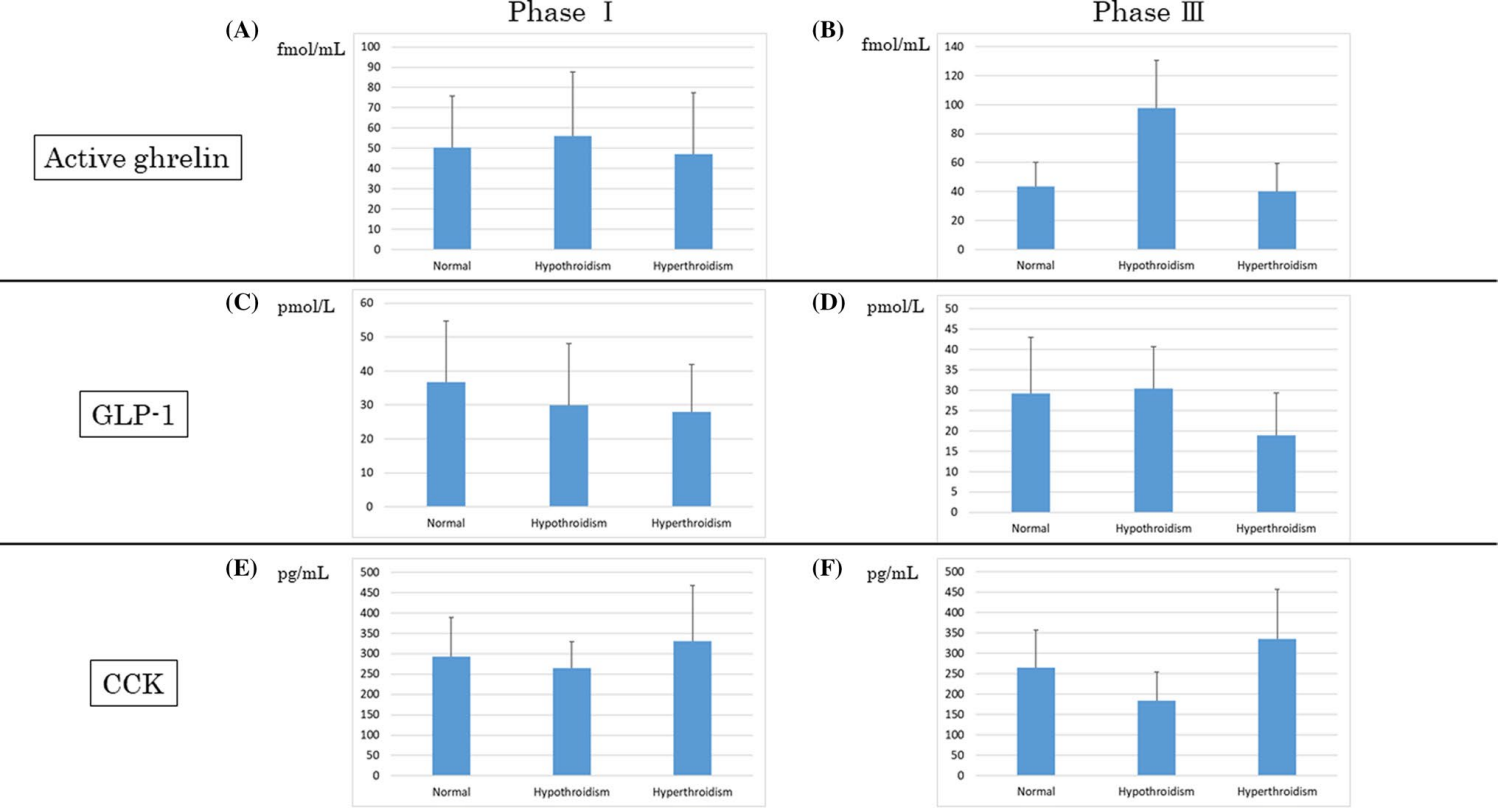
Plasma concentrations. (**A**) Active ghrelin, (**B**) GLP-1, and (**C**) CCK levels in phases I and III. However, there were no significant differences in the plasma concentrations under control, hypothyroid, and hyperthyroid states.

The plasma concentrations of ghrelin, GLP-1, and CCK in phases I and III under control, hypothyroid, and hyperthyroid states are presented in Fig. 4. The ghrelin level was highest in IMC phase I and lowest point in IMC phase III. However, the ghrelin concentration during phase III was higher than that during phase I due to hypothyroidism. However, there were no significant changes in GLP-1 and CCK levels.

## Discussion

This report first showed the relationship between thyroid function and GI motility under an experimental condition in which the feedback of thyroid function was completely removed. In hyperthyroidism, the duration of IMC phase I was shorter, and that of phase II and phase III was significantly longer. Moreover, both the continuous duration of strong tetanic contraction at the antrum and the duration of phase III within 10 h from the first IMC after a meal significantly increased. By contrast, hypothyroidism caused opposite events. We examined the change in GI transit speed based on thyroid function. However, there were no differences. Hence, the varying duration of phases II and III rather than the GI transit speed was caused by the difference in symptoms based on thyroid function. This is the first study to clarify the cause of the difference in symptoms depending on thyroid function by focusing on the IMC phases. We also found that GMC occurred from the upper GI tract. We proposed that the development of GMC in the upper GI tract in hyperthyroidism may be the cause of vomiting and diarrhea. In terms of GI hormones including ghrelin, GLP-1, and CCK, we evaluated each GI hormone during IMC phases I and III in control, hypothyroid, and hyperthyroid states. However, no significant results were obtained in this study. These findings indicated that the changes in the nerve and smooth muscles were more dependent on thyroid function than on GI hormone. We cannot implant force transducers in humans because of medical ethical problems and the need for invasive treatment. This is a very significant study that clarified the effect of thyroid function on GI motility by creating a quantitative and objectively observable model.

In this study, the duration of IMC phase I was shorter, and the frequency and duration of IMC phase III and strong tetanic contraction at the antrum were significantly longer in hyperthyroid states than in control and hypothyroid conditions. The duration of phase II in hyperthyroidism states was significantly longer than that in hypothyroid states. By contrast, there was no change in the transmission speed in any condition, and the characteristic symptom (diarrhea) in hyperthyroidism states was represented by IMC rather than the transmission speed. The duration of phase I was significantly longer in hypothyroidism states than in control and hyperthyroid states. This was considered a cause of constipation. In previous reports, the food transit time decreased due to hyperthyroidism, and this was attributed to the difference in the frequency of phase III rather than transmission speed^6–12^. In our study, we have expanded the measurement from the stomach to the jejunum. The administration of thyroid hormone decreased the MI in the fornix and gastric body and increased GI motility in the gastric antrum. These results indicate that decreased GI motility in the fornix and gastric body caused nausea and vomiting in hyperthyroidism states.

GMC has been observed in the small intestine after radiation treatment and is believed to be a cause of diarrhea^46^. In previous reports, GMC was detected in the small intestine upon the administration of thyroid hormone^1^. This study first reported the time that GMC was generated from the upper GI tract including in the stomach. One GMC occurred from the anal sides and propagated to the cardiac sides, and this propagation was similar to the emesis-like interdigestive motor activity. The other GMC occurred from the oral sides and propagated to the anal sides, and this was similar to the diarrhea-like interdigestive motor activity in our research. GMC was observed only during hyperthyroidism, which was considered a cause of both vomiting and diarrhea.

Regarding GI hormones, previous reports on ghrelin were controversial, and the difference in the results was attributed to variabilities in IMC phase^38–41^. In this study, no significant difference was observed in phases I and III. The ghrelin concentration was highest in phase I and was lowest in phase III. In the current study, in phase III, the ghrelin concentration in a hypothyroid state was higher than that in control and hyperthyroid states. Ghrelin weakens the function of motilin and suppresses IMC phase III. However, the ghrelin concentration in phase III in a hypothyroid state was higher than that in control and hyperthyroid states. There was no significant difference in the frequency and duration of phase III in hypothyroid state compared with the control condition. Thus, further examination should be conducted. The GLP-1 and CCK concentrations did not significantly differ in phases I and III. Diabetes mellitus and thyroid dysfunction are often observed in routine clinical practice, and they frequently coexist in patients. The mechanism of its occurrence is still not known. The examination of GLP-1, which is a glycemic control hormone, did not present with differences according to thyroid function. CCK also enhanced pancreatic secretory capacity. However, there was no difference in the current study. In summary, this study showed that GI hormones have minimal effect on the changes in GI motility in response to thyroid function.

What was the cause of the differences in GI motility based on the thyroid function? They may be attributed to varying GI hormone levels and autonomic nervous system function and changes in intestinal smooth muscle motility. However, as described above, no significant results on GI hormone levels were obtained, thereby indicating that these hormones may not be involved. Gaginella et al. reported that the receptor density and affinity of intestinal smooth muscle muscarinic receptors did not change significantly based on thyroid function^47^. By contrast, the administration of β-adrenergic blockers (sympathetic blockers) improved sympathetic nervous system activities in hyperthyroid patients with diarrhea^48^. In the study of intestinal smooth muscle, the morphology of the intestinal villi and the length and thickness of the intestinal wall changed according to thyroid function. In hypothyroidism, the intestinal villi were short and the intestinal wall (muscle layer) thickened, thereby increasing intestinal tone and leading to reduced intestinal motility^49,50^. Based on the abovementioned findings, the change in smooth muscle motility was considered a cause for the difference in GI motility. In this study, a series of experiments were performed on one dog under control, hypothyroid, and hyperthyroid states. Hence, histological examination could not be performed. However, changes in the sympathetic nervous system and intestinal smooth muscle itself caused the difference in GI motility.

This study had several limitations. That is, the number of dogs included in the experiment was small. However, since the results were reproducible, there was no need to increase the number of dogs, and the minimum number of required experiments was conducted from an ethical point of view. Moreover, histological examination can identify changes in the intestinal wall and GI hormone-secreting cells. However, this test was not performed. Hence, future studies should include histological examinations.

In conclusion, there was a relationship between thyroid function and GI motility in conscious dogs. In this study, we established an experimental condition that completely excluded the feedback. This study first reported that thyroid hormone activated upper GI motility without mediating GI hormones. Moreover, the administration of thyroid hormone induced both peristaltic and reverse peristaltic GMC. The difference in GI motility based on thyroid function and the cause of GI symptoms were also considered.

## Methods

### Animal experimentation

Three healthy female beagles weighing 8–10 kg were included in this study. All procedures were approved by the Animal Care and Experimentation Committee, Gunma University, Maebashi, Japan. Our experimental plan is presented in Fig. 1A. The dogs fasted overnight. Then, they were anesthetized with a single intravenous injection of thiopental sodium (Ravonal; Tanabe Pharmaceutical, Osaka, Japan) at a dose of 20 mg/kg. General anesthesia was maintained via intratracheal inhalation of halothane (Fluothane; Takeda Chemical Industries, Osaka, Japan) and oxygen. Under aseptic conditions, the Silastic tube (model 602–205; Dow Corning, Midland, MI) was inserted into the superior vena cava via a branch of the right external jugular vein (jugular catheter). Furthermore, it was used to withdraw blood samples and administer medications. The jugular catheter was exteriorized via a skin incision on the neck and was fixed to the adjacent skin with silk sutures. After the abdominal cavity was opened via a middle incision, force transducers^51,52^ were implanted onto the serosal surfaces of the fornix, gastric body, gastric antrum, midduodenum, and jejunums 1 and 2 (20 and 40 cm distal to the Treitz’s fascia, respectively) to detect circular muscle contraction. A force transducer is a contraction sensor that is sutured directly to a living organ, and it can directly detect GI motility in a natural state. In addition, in relation to nerves, hormones, and various accompanying organs, GI motility can be represented by a sharp waveform, which is useful in developing treatment methods and in determining the efficacy of new drugs.

The lead wires of the force transducers and the Silastic tubes were taken out of the abdominal cavity via a subcutaneous tunnel and were exteriorized via a skin incision made between the scapulae. After closure of the abdominal cavity, a jacket-type protector was placed on each dog to prevent the lead wires and tubes from being damaged when scratched by dogs themselves. The dogs were housed in individual experimental cages, maintained with intravenous drip infusions of Lactec G (Otsuka Pharmaceutical, Tokyo, Japan) for 5 days after surgery, and gradually returned to a normal chow diet (15 g/kg per day; Funabashi Farm, Funabashi, Japan). We waited 2 weeks for the dogs to recover and to present with IMC after the surgeries. Then, total thyroidectomy was performed. The anesthesia method described above was used. A 5-cm skin incision was made in the anterior neck. The parathyroid gland was preserved, and both the thyroid lobes were resected. Day 0 was defined as the day when total thyroidectomy was performed. Next, the dogs returned to a normal chow diet at day 1. The half-life of thyroid hormone in dogs is 10–16 h^53^. Hence, we performed an evaluation from days 1 to 4 in a hypothyroid state.

The production rate of total thyroxine (T4) in dogs is approximately 8 μg/kg/day^54,55^. Then, l-thyroxine sodium salt pentahydrate (Fujifilm Wako Pure Chemical Corporation, Osaka, Japan) was administered at a dose of 200 μg/kg/day after the patients were evaluated for hypothyroid state. The medication was continuously administered, and the evaluation of hyperthyroid state was again performed.

### Drug preparation

L-thyroxine sodium salt pentahydrate was prepared on each day, and it was dissolved in 200 μl of 1 N sodium hydroxide solution and diluted with 600 μl of water.

### Evaluation of thyroid function

We collected blood samples, as shown in Fig. 1A. In total, 1 mL of blood was collected in one blood collection. Blood samples were immediately transferred into test tubes containing a serum separating agent and were centrifuged at 4 °C at 3,000 rpm for 5 min. The plasma samples were sent to outsourced laboratory (FUJIFILM VET Systems Company Limited, Tokyo, Japan). The concentration of TSH, T4, and calcium (Ca) was then assessed. Ca was measured to confirm if the parathyroid gland was preserved. The results were confirmed within 1 day after sending, and thyroid function was confirmed each time.

### Monitoring of gastrointestinal motility contractions

The motility index (MI) was also assessed, as shown in Fig. 1A. The wires from the transducer were connected to a telemeter, and the data were transmitted to a recording system (Eight Star System, Star Medical, Tokyo, Japan). The recorded signals were used to identify each phase of the contractile activity and the MI. The MI was the integrated area between the baseline (zero level) and the contractile wave on the Eight Star System.

We compared the first IMC that appeared after a meal within 5 h with GI according to thyroid state. Moreover, the MI 2 h before and after thyroid hormone was administered in a hyperthyroid state was calculated.

### Evaluation of transmission speed

The distance between jejunums 1 and 2 was 20 cm. The time difference between the start of IMC phase III in jejunums 1 and 2 was evaluated, and the transmission speed (cm/second) was calculated.

### Measurement of active ghrelin, GLP-1, and CCK levels

We collected blood samples, as shown in Fig. 1A. The samples were obtained during phases III and I. In total, 2 mL of blood was collected in one blood collection. All blood samples were placed into chilled tubes containing ethylenediaminetetraacetic acid-2Na and 500 U apoprotein and were centrifuged at 4 °C at 3,000 rpm. Two plasma samples were immediately collected. One was used for active ghrelin measurement, and the other for GLP-1 and CCK measurement. To measure active ghrelin using the enzyme-linked immunosorbent assay (ELISA), 0.1 mL of 1 N hydrochloric acid was added to per 1 mL of the samples. All samples were stored at − 80 °C until hormone concentration analyses. The plasma active ghrelin concentrations were measured using the ELISA kit (Life Science Institute, Inc., Tokyo, Japan). The plasma GLP-1 concentrations were evaluated using the canine ELISA kit (MyBioSource, Inc., San Diego, California, the USA). Moreover, the plasma CCK concentrations were assessed using the canine ELISA kit (Antibodies.com., Cambridge, the UK).

### Statistical analysis

The results were expressed as mean ± standard error. The data were compared using the paired *t*-test (Tukey’s test). *P* values < 0.05 were considered statistically significant. All statistical analyses were performed using the JMP software (SAS Institute Inc., Cary, NC, the USA).

### Ethical perspective

Because the data were consistent, the experiment was performed on three conscious dogs. The study was performed in accordance with both the Animal Welfare and the International Guiding Principles for Biomedical Research Involving Animals^56^. All procedures were approved by the Animal Care and Experimentation Committee, Gunma University, Maebashi, Japan (approval no. 17-045). The study was carried out in compliance with the ARRIVE guidelines.

### Ethics declaration

The study was performed in accordance with both the Animal Welfare and the International Guiding Principles for Biomedical Research Involving Animals.

### Approval of animal experiments

All procedures were approved by the Animal Care and Experimentation Committee, Gunma University, Maebashi, Japan (approval no. 17-045).

## Data Availability

The datasets generated during and/or analyzed during the current study are available from the corresponding author on reasonable request.
